# Dual Role of Gibberellin in Perennial Shoot Branching: Inhibition and Activation

**DOI:** 10.3389/fpls.2020.00736

**Published:** 2020-06-05

**Authors:** Niveditha Umesh Katyayini, Päivi L. H. Rinne, Danuše Tarkowská, Miroslav Strnad, Christiaan van der Schoot

**Affiliations:** ^1^Department of Plant Sciences, Norwegian University of Life Sciences, Ås, Norway; ^2^Laboratory of Growth Regulators, Faculty of Sciences, Institute of Experimental Botany of the Czech Academy of Sciences, Palacký University Olomouc, Olomouc, Czechia

**Keywords:** gibberellin, axillary branching, GA2-oxidases, GA3-oxidases, GA20-oxidases, GID1, strigolactone, hormones

## Abstract

Shoot branching from axillary buds (AXBs) is regulated by a network of inhibitory and promotive forces, which includes hormones. In perennials, the dwarfed stature of the embryonic shoot inside AXBs is indicative of gibberellin (GA) deficiency, suggesting that AXB activation and outgrowth require GA. Nonetheless, the role of GA in branching has remained obscure. We here carried out comprehensive GA transcript and metabolite analyses in hybrid aspen, a perennial branching model. The results indicate that GA has an inhibitory as well as promotive role in branching. The latter is executed in two phases. While the expression level of *GA2ox* is high in quiescent AXBs, decapitation rapidly downregulated it, implying increased GA signaling. In the second phase, *GA3ox2*-mediated *de novo* GA-biosynthesis is initiated between 12 and 24 h, prior to AXB elongation. Metabolite analyzes showed that GA_1/4_ levels were typically high in proliferating apices and low in the developmentally inactive, quiescent AXBs, whereas the reverse was true for GA_3/6_. To investigate if AXBs are differently affected by GA_3_, GA_4_, and GR24, an analog of the branch-inhibitor hormone strigolactone, they were fed into AXBs of single-node cuttings. GA_3_ and GA_4_ had similar effects on GA and SL pathway genes, but crucially GA_3_ induced AXB abscission whereas GA_4_ promoted outgrowth. Both GA_3_ and GA_4_ strongly upregulated *GA2ox g*enes, which deactivate GA_1/4_ but not GA_3/6_. Thus, the observed production of GA_3/6_ in quiescent AXBs targets GA_1/4_ for GA2ox-mediated deactivation. AXB quiescence can therefore be maintained by GA_3/6_, in combination with strigolactone. Our discovery of the distinct tasks of GA_3_ and GA_4_ in AXB activation might explain why the role of GA in branching has been difficult to decipher. Together, the results support a novel paradigm in which GA_3/6_ maintains high levels of *GA2ox* expression and low levels of GA_4_ in quiescent AXBs, whereas activation and outgrowth require increased GA_1/4_ signaling through the rapid reduction of GA deactivation and subsequent GA biosynthesis.

## Introduction

Shoot branching is governed by a network of hormones that includes auxin, cytokinin (CK) and strigolactone (SL). How they interact to regulate axillary bud (AXB) activation and outgrowth still divides opinion (Ferguson and Beveridge, 2009; Hayward et al., 2009; Müller and Leyser, 2011; Puig et al., 2012; Rameau et al., 2015). Classic experiments established that a growing shoot apex can repress branching, a phenomenon known as apical dominance. The physiological explanation is that a proliferating apex produces a surplus of auxin that is send down the stem to inhibit AXB outgrowth, thereby promoting apical elongation. Removal of the apex releases AXBs from inhibition, triggering branching, but this can be prevented by supplying auxin to the cut stem (Thimann and Skoog, 1934; Phillips, 1975; Cline, 1991, 1997).

A current interpretation of these experiments is that the growing apex increases the relative amount of auxin in the polar auxin transport stream (PATS) of the main stem, thereby preventing AXBs from establishing their own auxin export path to the stem (Li and Bangerth, 1999; Bennett et al., 2006; Ongaro et al., 2008; Domagalska and Leyser, 2011). When auxin levels in the stem drop, export of auxin from the AXB to the stem is initiated, promoting AXB outgrowth. An alternative model proposes that auxin export is a consequence of AXB activation rather than a cause (Dun et al., 2006; Brewer et al., 2009; Ferguson and Beveridge, 2009). This is in line with the proposal of Cline (1997) that a fast initial enlargement of an AXB should be distinguished from the much slower outgrowth process. Experimental support comes from studies with garden pea (*Pisum sativum* L.), in which shoot decapitation triggers AXB enlargement ahead of the arrival of the auxin depletion front (Morris et al., 2005). Moreover, supplying auxin to the cut stem can prevent branching but not AXB enlargement. Finally, depleting stem auxin levels by auxin transport inhibitors does not affect initial AXB enlargement, but once AXBs have enlarged it promotes sustained outgrowth (Morris et al., 2005; Ferguson and Beveridge, 2009; Mason et al., 2014). In addition to the network of hormones, nutrients are important in AXB outgrowth in intact plants, as well as after decapitation when sugars are diverted to the larger AXBs, which are the strongest sinks (Mason et al., 2014; Kebrom, 2017).

The transcription factor BRANCHED1 (BRC1)/TEOSINTE BRANCHED1 (TB1) is an important branch-inhibitor (Aguilar-Martínez et al., 2007; Brewer et al., 2009; Dun et al., 2009; Leyser, 2009). Although *BRC1* was originally identified as the target of SL, it is now recognized to be a hub for branch-regulating signals, including various hormones and developmental as well as environmental cues (Wang et al., 2019). In Arabidopsis (*Arabidopsis thaliana*), BRC1 inhibits AXB outgrowth, probably by suppressing cell proliferation (Schommer et al., 2014), but in some circumstances it cannot prevent outgrowth (Seale et al., 2017). In rice (*Oryza sativa*), SL also induces degradation of the branch-promoting hormone CK through transcriptional activation of CK-oxidases (Duan et al., 2019). In accordance with this, AXB outgrowth in pea is accompanied by a reduction in SL biosynthesis and an increase in CK biosynthesis (Tanaka et al., 2006; Ferguson and Beveridge, 2009). Auxin also suppresses CK biosynthesis (Nordström et al., 2004). Thus, CK-induced outgrowth of activated AXBs may require low stem levels of auxin and SL.

While auxin, CKs and SLs are implicated in the regulation of AXBs, the role of gibberellins (GA) has remained obscure. This is unexpected as GAs promote many developmental processes, including germination, elongation, floral transition as well as AXB formation and dormancy release (Hazebroek et al., 1993; Richards et al., 2001; Yamaguchi, 2008; Rinne et al., 2011, 2016; Claeys et al., 2014; Zhuang et al., 2015). GA is often viewed as a branch-inhibitor because GA-biosynthesis and -perception mutants in Arabidopsis, as well as GA-deficient transgenic plants of various species have branched phenotypes. However, a complicating factor is that GA-deficiency or lack of GA perception not only increases branching but also reduces apical dominance (Scott et al., 1967; Talon et al., 1990; Murfet and Reid, 1993; Silverstone et al., 1997; Olszewski et al., 2002; Busov et al., 2003; Agharkar et al., 2007; Lo et al., 2008; Mauriat et al., 2011; Zawaski and Busov, 2014; Rameau et al., 2015). In contrast to the above, some studies suggest that GA promotes branching. In perennial strawberry, AXB outgrowth is diminished in a GA-biosynthesis mutant, while GA supply rescues the phenotype (Tenreira et al., 2017). Similarly, in the woody species *Jatropha* (*J. curcas* L.) (Ni et al., 2015) and hybrid aspen (*Populus tremula* × *P. tremuloides*) (Rinne et al., 2011), GA application promotes AXB outgrowth, whereas in *Rosa* sp. outgrowth requires GA biosynthesis (Choubane et al., 2012).

**FIGURE 1:**
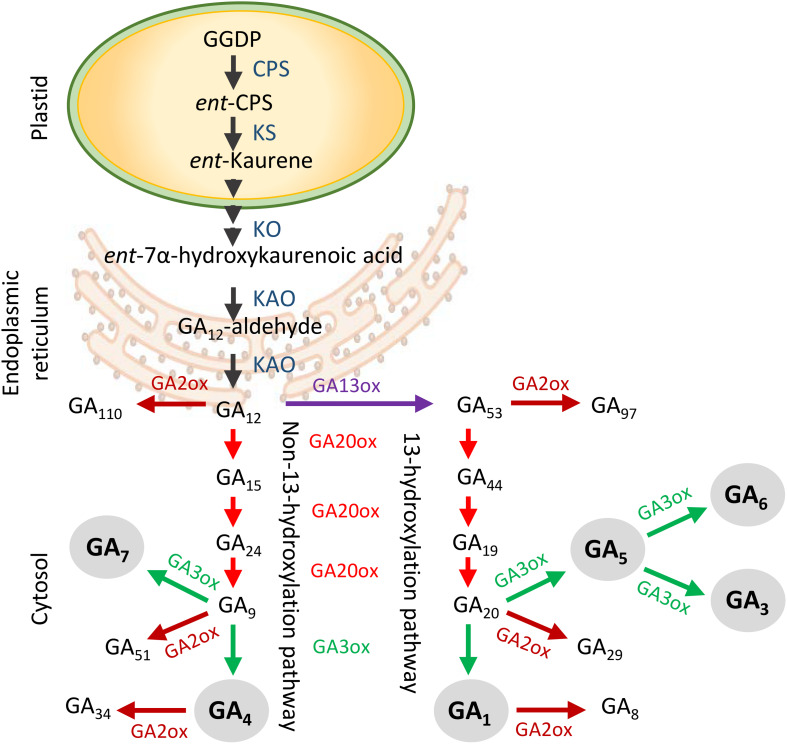
Generalized scheme of gibberellin (GA) biosynthesis and deactivation in higher plants. GA biosynthesis starts in the plastids and is followed by the production of GA_12_ in the endoplasmic reticulum. In the cytoplasm GA_12_ is processed by GA20ox and GA3ox enzymes in two separate branches to produce bioactive GAs (gray circles). The non-13-hydroxylation yields GA_7_ and GA_4_, whereas the 13-hydroxylation yields GA_5_, GA_6_, GA_3_, and GA_1_. The GA2ox enzymes deactivate precursors and bioactive GAs. Abbreviations: GGDP, geranylgeranyl diphosphate; CPS, *ent-*copalyl diphosphate synthase; KS, *ent*-kaurene synthase; KO, *ent*-kaurene oxidase; KAO, *ent*-kaurenoic acid oxidase.

Only a small number of the more than 130 known GAs is biologically active, including GA_1_, GA_3_, GA_4_, GA_5_, GA_6_, and GA_7_ (King et al., 2001, 2003; Yamaguchi, 2008; Hedden and Sponsel, 2015). GA biosynthesis starts with plastid-localized geranylgeranyl diphosphate (GGDP), which is converted to *ent-*kaurene (Figure 1; Hedden and Phillips, 2000; Olszewski et al., 2002; Yamaguchi, 2008), and oxidized by cytochrome P450 mono-oxygenase in the endoplasmic reticulum to yield GA_12_ (Helliwell et al., 2001). From there, metabolites are shuttled through two parallel cytoplasmic pathways, the non-13-hydroxylation and 13-hydroxylation pathway, in which three groups of 2-oxoglutarate-dependent dioxygenases provide catalytic activity (Hedden and Phillips, 2000; Yamaguchi and Kamiya, 2000; Olszewski et al., 2002; Hedden and Thomas, 2012). These include GA20-oxidases (GA20oxs) that produce GA precursors, GA3-oxidases (GA3oxs) that produce bioactive GAs, and GA2-oxidases (GA2oxs) that irreversibly deactivate precursors and bioactive GAs by 2β-hydroxylation (Thomas et al., 1999; Lo et al., 2008; Rieu et al., 2008a). Which of the two pathways is dominant depends on species, developmental stage, and organ type. For example, in rice, GA_1_ dominates during vegetative growth but during anthesis it is GA_4_ (Kobayashi et al., 1989; Hirano et al., 2008), whereas in hybrid aspen GA_4_ regulates shoot elongation (Israelsson et al., 2004) and in Arabidopsis also flowering (Sponsel et al., 1997; Eriksson et al., 2006). GA signaling requires binding to the receptor GIBBERELLIN INSENSITIVE DWARF1 (GID1), which localizes to the cytoplasm and nucleus (Ueguchi-Tanaka et al., 2005; Willige et al., 2007; Hirano et al., 2008; Sun, 2010). Because GA_4_ has the highest affinity to GID1 (Nakajima et al., 2006), its effective concentration can be low. GA-GID1 binding enhances interaction with growth-repressor DELLA proteins, which are also present in the cytoplasm and nucleus (Sun, 2011; Davière and Achard, 2013). Subsequent interaction with ubiquitin E3 ligase complex SCF^SLY1/GID2^ leads to ubiquitination and degradation of DELLA (Peng et al., 1997; Silverstone et al., 1998; Bolle, 2004; Ueguchi-Tanaka et al., 2007).

It is uncertain if the herbaceous branching models can be transferred directly to woody perennials, considering their different shoot size, lifespan, and AXB composition. In hybrid aspen nodal bark tissue might contribute to the regulation of AXB behavior, perhaps compensating for the inefficiency of long-distance transfer of root-produced strigolactone precursors (Katyayini et al., 2019). In hybrid aspen, AXBs are elaborate structures with sturdy scales that enclose a dwarfed embryonic shoot (ES) that arises over a developmental time span of 10 to 12 plastochrons (Rinne et al., 2015). However, deciduous perennials can show strikingly distinct branching styles, suggesting that even within them regulation of AXB outgrowth can differ. In sylleptic species, AXBs grow out in the same season, producing plastic branching patterns in response to environmental conditions (Wu and Stettler, 1998; Wu and Hinckley, 2001), whereas in proleptic species AXBs do not grow out in the same year (Hallé et al., 1978; Barthélémy and Caraglio, 2007). In hybrid aspen, AXBs cease development at the bud maturation point (BMP) and remain inactive until the next growing season (Paul et al., 2014). The AXBs can therefore be viewed as containing side shoots in which phytomer development is temporarily decoupled from stem elongation, which is postponed until the next growing season. In spring, the elongating stem of the ES telescopes out of the opening bud, allowing subsequent neoformation of leaves. Despite being locked in a developmentally quiescent state, the current year AXBs have a high potential for outgrowth, as shoot decapitation induces rapid outgrowth.

Previous analyses of several GA pathway genes in hybrid aspen suggested that GA-deficiency could explain the dwarfed nature of the ES, and that GA biosynthesis would be required for decapitation-induced elongation (Rinne et al., 2015, 2016). ES elongation might require GA_4_ to regulate cell division and cell stretching, and to recruit GA_4_-inducible 1,3-β-glucanases that optimize symplasmic conduits for nutrient and sugar import (Rinne et al., 2011, 2016). While different GA forms can have different developmental effects, the basis of this has not been investigated. To our knowledge, it has remained unknown which GAs play a role during AXB quiescence and branching in hybrid aspen as well as other woody perennial species. The relative prominence of AXBs in hybrid aspen permitted us to carry out comprehensive analyzes of GA metabolite levels and GA-pathway transcripts.

The results support a novel paradigm of a dual role of GA in shoot branching, in which GA_3/6_ and GA_1/4_ have opposing tasks. AXBs produce GA_3/6_ to maintain quiescence by upregulating *GA2ox* genes, which deactivate GA_1/4_, keeping their levels low despite ongoing biosynthesis. AXB activation, in turn, is achieved by the instantaneous and strong downregulation of the *GA2ox* genes, boosting GA_1/4_-induced signaling. Subsequent elongation is followed by GA_1/4_ biosynthesis through *GA3ox2* and supported by GA precursor import from the node.

## Results

To understand the role of GA in shoot branching, we mapped the expression of all GA pathway genes in the major parts of intact plants, and in decapitation activated AXBs and associated nodes. The data were combined with analyses of GA intermediates and bioactive GAs. As GA and SL are thought to have opposite effects on AXB activation, we investigated how feeding of GA_3_, GA_4_ and the synthetic SL analog GR24 into AXBs of single-node cuttings influenced the expression of GA and SL pathway genes.

### *GA20oxs* and *GA3oxs* Expression Is Organ- and Development-Related

**FIGURE 2:**
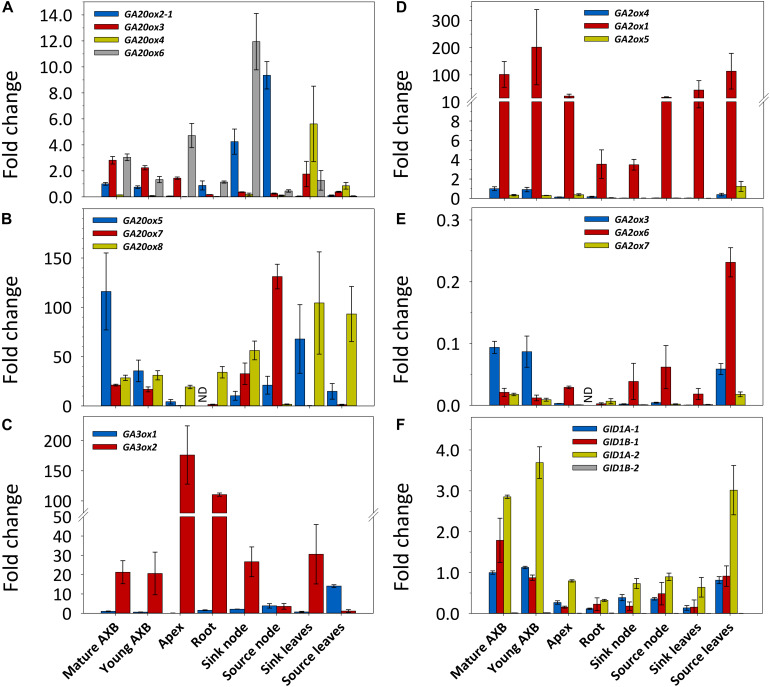
Expression of gibberellin (GA) biosynthesis, deactivation and signaling genes in different plant parts in hybrid aspen. Relative expression (fold change) of *GA20ox* **(A,B)**, *GA3ox* **(C)**, *GA2ox* **(D,E)**, and *GID1* **(F)** family genes. The two larger gene families are depicted in two separate graphs with high **(B,D)** and little **(A,E)** expressed genes. Values represent the means of three biological replicates ± S.E. (*n* = six plants). ND, not detected. Fold changes are relative to reference gene expression in quiescent AXBs, set to 1. A moderately expressed gene within each family was selected for comparison: *GA20ox2-1*, *GA3ox1*, *GA2ox4*, and *GID1A-1*.

The genome of *P. trichocarpa* contains eight *GA20ox* and three *GA3ox* genes (Tuskan et al., 2006; Figure 1 and Supplementary Figure S1), but preliminary studies showed that *GA20ox2-2* was not expressed in hybrid aspen. Transcripts of the seven remaining *GA20ox* genes were present in young (developing) and mature (developmentally quiescent) AXBs (Figure 2). In decreasing order, the highest transcript levels were found for *GA20ox5*, *GA20ox8* and *GA20ox7*, whereas *GA20ox6*, *GA20ox3* and *GA20ox2-1* were little expressed, and *GA20ox4* only in leaves (Figures 2A,B). Of the highly expressed genes of this family, *GA20ox8* was the most generally expressed, but transcript levels were especially high in leaves. Whereas in bark tissue of nodes associated with sink leaves (denoted sink nodes) *GA20ox8* expression was high, it was almost completely absent in bark tissue of nodes at source leaves (denoted source nodes). Except for the AXBs, all other plant parts expressed *GA20ox* genes selectively, suggesting the various paralogs might have tissue-specific roles. That all *GA20ox* family genes were expressed in AXBs makes sense as AXBs harbor a complete, albeit dwarfed shoot system. Combining the transcript levels of all *GA20ox* paralogs showed that GA-precursor production was highest in sink leaves, followed by source nodes and associated AXBs. In contrast, roots and apices had low transcript levels (Figures 2A,B). Although transcript levels in young AXBs were approximately half of those in the mature quiescent AXBs, they were still almost three times higher than in apices and root tips.

Mature as well as young developing AXBs expressed *GA3ox1* and *GA3ox2*, but transcript levels of *GA3ox1* were significantly lower than those of *GA3ox2* (Figure 2C and Supplementary Figure S1). In apices, *GA3ox1* was virtually absent, whereas it increased in nodes and leaves during their maturation, reaching the highest levels in source leaves. In stark contrast, the expression of *GA3ox2* was very high in proliferating shoot apices, and high in growing root tips, sink nodes, sink leaves and AXBs. In the mature nodes and leaves *GA3ox2* expression was considerably reduced. The expression ratio of *GA3ox2*/*GA3ox1* showed that apices had the highest approximate ratio (1000), followed by tissues in sinks (20) and sources (0.25). Together the results reveal that, rather than being tissue specific, *GA3ox1* and *GA3ox2* are developmentally regulated, and that their physiological importance is reversed during tissue maturation. Thus, *GA3ox2* expression supports cell proliferation and growth, whereas *GA3ox1* is dominant in mature tissues.

In summary, the spatio-temporal expression patterns of the *GA20ox* and *GA3ox* family members show that source nodes and source leaves might stockpile GA precursors for delivery to AXBs, while AXBs themselves can produce precursors as well as bioactive GAs.

### *GA2ox* Gene Expression Is Highest in AXBs and Source Leaves

In *P. trichocarpa*, the GA-deactivating *GA2ox* family is composed of seven genes (Gou et al., 2011; Supplementary Figure S1). *GA2ox2* was not expressed at measurable amounts in shoot tissues of hybrid aspen (not shown) and therefore was not included in the analyses. In decreasing order, the highest transcript levels were found for *GA2ox1*, *GA2ox4*, *GA2ox5, GA2ox6, GA2ox3*, and *GA2ox7* (Figures 2D,E). In all plant parts, *GA2ox1* had by far the highest transcript levels of the entire *GA2ox* family. The little expressed genes, *GA2ox5* and *GA2ox6*, were most highly expressed in source leaves. AXBs and source leaves stood apart by expressing most genes, and having the highest combined expression levels, around six times more than apices. Notably, the actively growing tissues, including apices, sink nodes and sink leaves, which expectedly are most active in GA signaling, all expressed *GA2ox* genes at a low level.

### *GID1* Receptor Gene Expression Is Highest in AXBs

We identified in hybrid aspen all four paralogs of the *P. trichocarpa GID1* genes, and named them *GID1A-1*, *GID1A-2*, *GID1B-1*, and *GID1B-2* (Supplementary Figure S2). In shoot tissues, transcript levels of *GID1A-2* were the highest, followed by *GID1B-1* and *GID1-A1*, whereas expression of *GID1B-2* was very low (Figure 2F). The combined transcript levels of *GID1* genes were clearly highest in AXBs and source leaves. In contrast, the expression was low in strong sinks, including proliferating apices, growing root tips, sink nodes and sink leaves. As growing tissues, but especially apices, expressed high levels of the proliferation-related GA-biosynthesis gene *GA3ox2* (Figure 2C), the lower *GID1* expression levels are expected to reflect high levels of bioactive GAs because receptor abundance correlates negatively with GA levels (Middleton et al., 2012).

### *GA20ox* and *GA3ox* Genes Are Not Early Activators of AXBs

Even though AXBs in hybrid aspen become quiescent when they reach the BMP, they maintained elevated transcript levels of *GID1* receptor genes (Figure 2F), indicating that they remain highly sensitive to GA even after completing development. Despite this, AXBs forestall outgrowth, likely through high expression of *GA2ox* genes to neutralize GA biosynthesis (Figures 2D,E).

**FIGURE 3:**
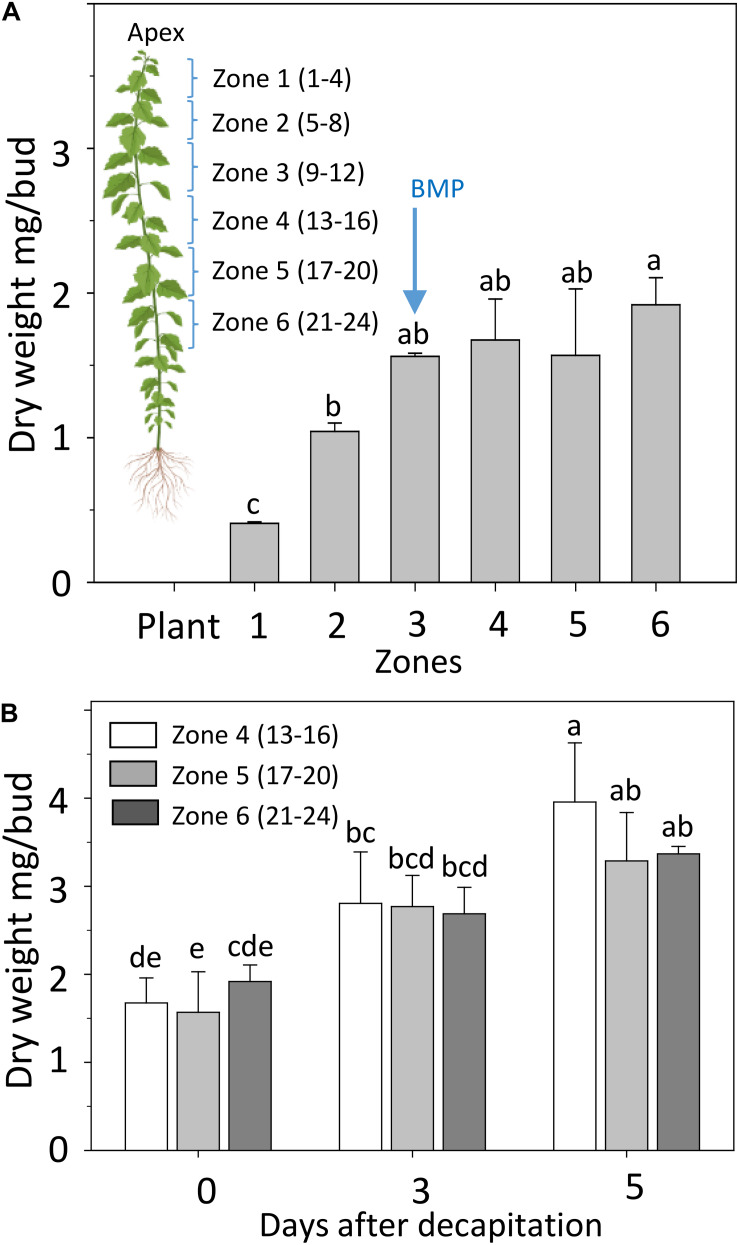
Development of AXBs. **(A)** AXBs in different zones along the stem in intact plants. The numbers in parenthesis of zones indicate the position of AXBs, counted from the top. **(B)** AXB enlargement after decapitation in the remaining zones 4–6. Values represent the means of three biological replicates ± S.E. (*n* = six plants). One-way ANOVA. Different letters indicate statistical differences between samples (Fischer’s LSD *post hoc* analysis; *P*-value at least < 0.05).

To investigate if and how deactivation and GA biosynthesis changes during AXB activation and outgrowth we decapitated plants at the BMP, recorded the growth of the proximal AXBs over a 5-day period, and analyzed the changes in gene expression that occurred during the critical first 48 h. The BMP has been assessed before, based on the number of embryonic leaves (Rinne et al., 2015). Here, we determined the AXB growth by monitoring the dry weight increment of AXBs along the stem. A plateau in weight gain was reached at the end of zone 3, which is around AXB 12 (Figure 3A), in agreement with the earlier assessment based on embryonic leaf number (Rinne et al., 2015). Precise weight measurements revealed that decapitation not only significantly increased the weight of the proximal AXBs (zone 4), but also of the lower AXBs (zone 5 and 6), showing that all AXBs were activated (Figure 3B). Nonetheless, only the uppermost AXBs (zone 4) grew out, indicating that AXB activation is distinct from outgrowth, as suggested earlier (Cline, 1997).

**FIGURE 4:**
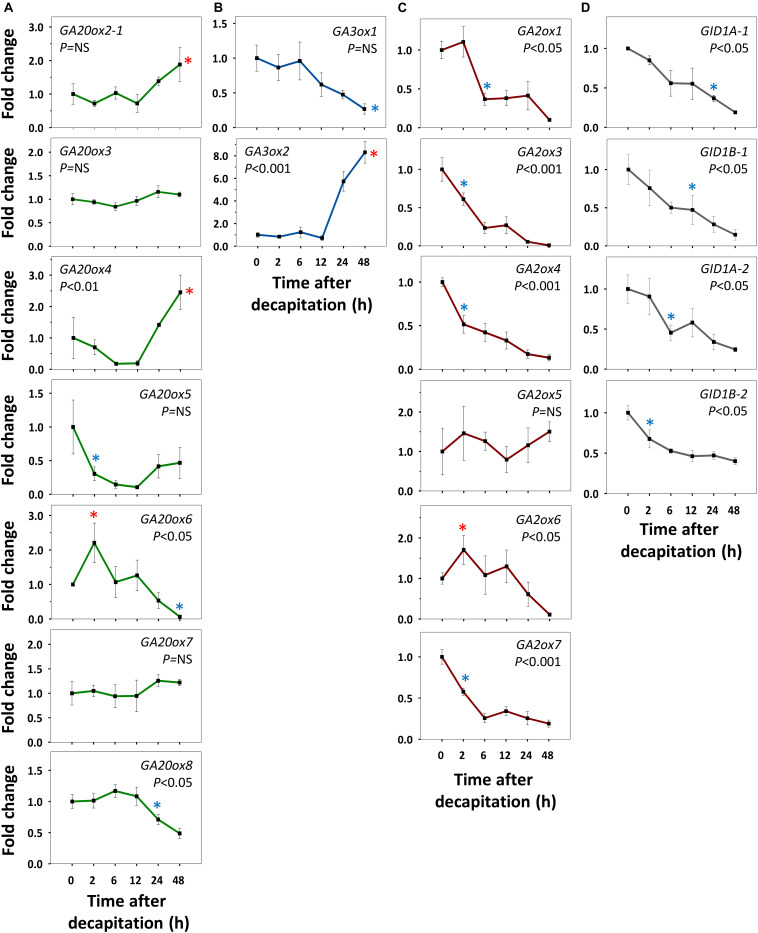
Expression of gibberellin (GA) biosynthesis, deactivation and signaling genes in AXBs after activation by decapitation. **(A)** GA biosynthesis gene families encoding GA20-oxidases and **(B)** GA3-oxidases. **(C)** GA-deactivation gene family encoding GA2-oxidases. **(D)** GID1-receptor genes. Relative expression (fold change) was analyzed at indicated times after decapitation in three successive AXBs proximal to the decapitation point. Values represent the means of three biological replicates ± S.E. (*n* = six plants). One-way ANOVA (*P*-value; NS, not significant). Asterisk indicates the first significant decrease (blue) or increase (red), in gene expression in comparison to time 0 (Fischer’s LSD *post hoc* analysis, *P*-value at least < 0.05).

Refining our previous suggestion (Rinne et al., 2016), we show here that net GA-biosynthesis is not the first step in decapitation-induced AXB activation. Although *GA20ox6* increased transiently at 2 h, and *GA20ox2-1* and *GA20ox4* at 48 h, these genes were little expressed in quiescent AXBs compared to *GA20ox5* and *GA20ox8*, which significantly decreased by 2 and 24 h, respectively (Figure 4A). Strikingly, the proliferation-related gene *GA3ox2*, serving *de novo* biosynthesis of GA, became significantly upregulated only between 12 and 24 h, in parallel with the downregulation of maturation-related *GA3ox1* (Figure 4B). In brief, *de novo* GA biosynthesis by *GA20ox* and *GA3ox* genes is not the initial factor that triggers AXB activation.

### *GA2ox* Genes Are Early Responders During AXB Activation

**FIGURE 5:**
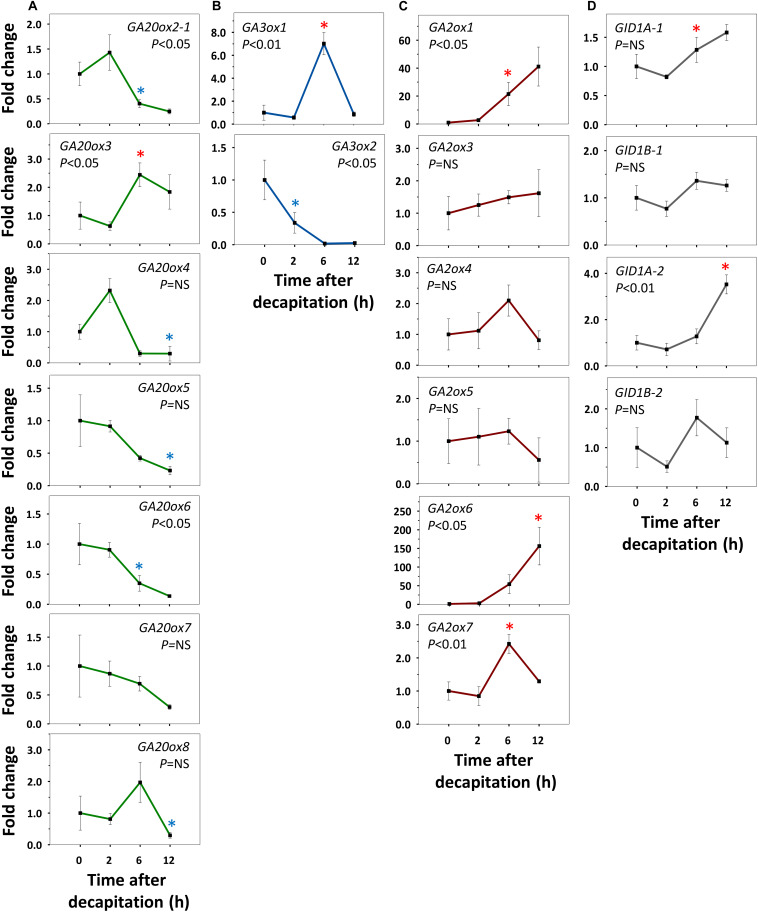
Expression of gibberellin (GA) biosynthesis, deactivation and signaling genes in nodal bark after decapitation. **(A)** GA biosynthesis gene families encoding GA20-oxidases and **(B)** GA3-oxidases. **(C)** GA-deactivation gene family encoding GA2-oxidases. **(D)** *GID1*-receptor genes. Relative expression (fold change) was analyzed at indicated times after decapitation in three successive nodes proximal to the decapitation point. Values represent the means of three biological replicates ± S.E. (*n* = six plants). One-way ANOVA (*P*-value; NS, not significant). Asterisk indicates the first significant decrease (blue) or increase (red), in gene expression in comparison to time 0 (Fischer’s LSD *post hoc* analysis, *P*-value at least < 0.05).

All AXBs of intact plants expressed the GA-deactivating *GA2ox* genes, some at relatively high or very high levels (Figures 2D,E), but decapitation significantly downregulated them within a few hours (Figure 4C). This represented the first change induced by decapitation. The highly expressed gene *GA2ox1* was strongly downregulated between 2 and 6h post-decapitation, whereas the little expressed genes *GA2ox3*, *GA2ox4*, and *GA2ox7* were downregulated even earlier (Figure 4C). The remaining two little-expressed genes *GA2ox6* and *GA2ox5* responded later or not at all. This shows that the considerable levels of *GA3ox1* and *GA3ox2* expression in quiescent AXBs were counteracted by the high levels of *GA2ox1* expression. In other words, deactivation neutralizes biosynthesis in quiescent AXBs, whereas decapitation increases bioactive GAs by strongly reducing GA deactivation (Figure 4C). The significant parallel reduction in the expression of the *GID1* genes (Figure 4D) supports this conclusion, as it is well-known that transcription of *GID1* is reduced when levels of bioactive GAs rise (Middleton et al., 2012). That the expression of *GA20ox* genes did not increase in AXBs after decapitation, while the expression of *GA3ox2* was significantly elevated at 24 h, may indicate that additional GA precursors arrived from the nodes. In support of this, expression of *GA20ox2-1*, *GA20ox5* and *GA20ox7* in source nodes was high (Figures 2A,B), and decapitation transiently upregulated *GA20ox2-1*, *GA20ox3*, *GA20ox4*, and *GA20ox8* (Figure 5A). The putative pool of precursors in the nodes is unlikely to serve the production of bioactive GA in the source node itself, because the proliferation-related gene *GA3ox2* was little expressed, and further downregulated 2 h post-decapitation (Figure 5B). Although the maturation-related gene *GA3ox1* was transiently upregulated in source nodes between 2 and 6 h (Figure 5B), this was offset by the dramatic upregulation of *GA2ox1* and *GA2ox6*, the two major deactivating genes, as well as the little-expressed gene *GA2ox7* (Figure 5C). Moreover, the expression of the *GID1* receptor genes tended to increase in the nodes, suggesting a reduction in bioactive GA levels. Notably, the expression patterns of GA-biosynthesis, GA-deactivation and *GID1* receptor genes were almost opposite in nodes and activated AXBs (Figures 4, 5).

Collectively, the results support the idea that nodal bark acts as a storage of GA precursors. The time frame of the events suggests that AXB activation is based on diminished deactivation of bioactive GAs in AXBs, making them available for GA signaling, whereas outgrowth relies on biosynthesis, assisted by delivery of node-produced GA precursors.

### Xylem-Fed GA_3_, GA_4_, and GR24 Modulate GA- and SL-Pathways

Although often functioning redundantly, GA_3_ and GA_4_ are produced in separate biosynthetic branches. A biologically meaningful distinction is that GA_4_ is deactivated by GA2oxs, whereas GA_3_ is protected by a double bond at the C2, preventing 2β-hydroxylation (Nakayama et al., 1990). In hybrid aspen, GA_4_ application to dormant AXBs triggers outgrowth, whereas GA_3_ fails to do so, and a high concentration induces AXB abscission (Rinne et al., 2011). Another factor that affects AXB activation is SL, which acts as an inhibitor of outgrowth in hybrid aspen (Katyayini et al., 2019).

**FIGURE 6:**
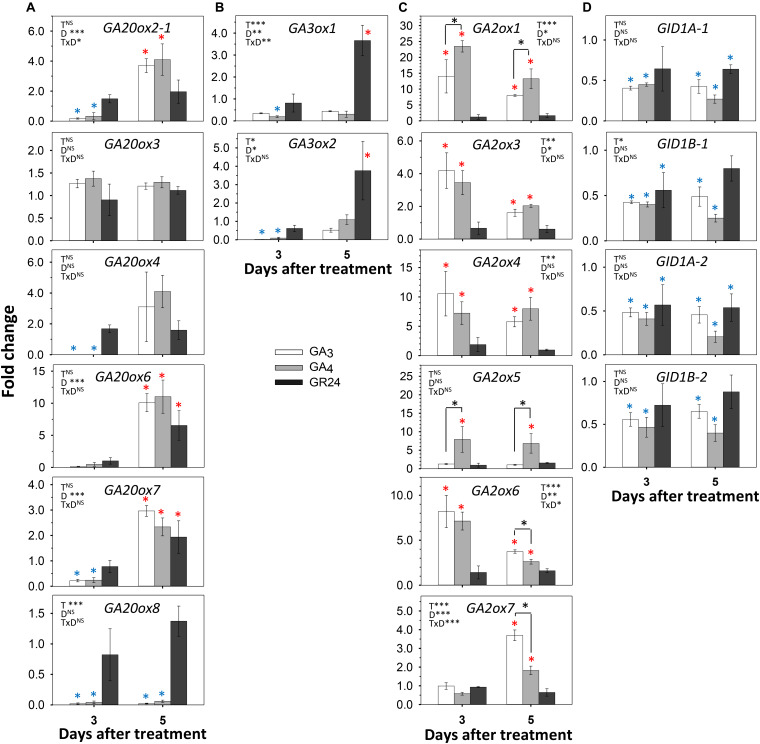
Effect of GA_3_, GA_4_, and GR24 on expression of GA-pathway genes in AXBs. AXBs on single-node cuttings were fed with or without GA_3_, GA_4_ and GR24 at a concentration of 10 μM. **(A)** *GA20-oxidase* genes. **(B)** *GA3-oxidase* genes. **(C)** *GA2-oxidase* genes. **(D)** *GID1* genes. Values are calculated relative to control and represent the means of three biological replicates ± S.E. (*n* = six plants). The significance of factors in two-way ANOVA (T, treatments; D, duration in days; TxD, interaction) are indicated by asterisk(s) (*P < 0.05; **P < 0.01; and ***P < 0.001). Asterisks above the bar indicate decrease (blue) or increase (red), relative to control, and above the hook differences between GA_3_- and GA_4_-treatments (Fischer’s LSD *post hoc* analysis; *P*-value at least < 0.05).

To investigate possible interference of these three hormone pathways, we fed them separately into single-node cuttings, monitored AXB behavior, and analyzed the expression of GA- and SL-pathway genes. As the simple act of isolating the single-node cuttings already activates the AXBs, these experiments test possible interference during AXB elongation. Because preliminary tests with 1% methylene blue showed that it took more than 24 h before dye entered AXBs (not shown), the analyses were carried out at day 3 and day 5, well within the AXB elongation phase. AXB outgrowth tests showed that feeding of a relatively high concentration of GR24 neither inhibited nor promoted AXB burst, relative to the controls, while GA_4_ significantly accelerated it, and GA_3_ induced AXB abscission (Supplementary Figure S3). Gene expression analyses of AXBs showed that at the 3 d time point *GA20ox* genes were downregulated by both GA_3_ and GA_4_, except for the unresponsive *GA20ox3*, and there was no clear difference between the effects of GA_3_ and GA_4_ (Figure 6A). At the 5 d time point, the downregulated *GA20ox2-1*, *GA20ox4, GA20ox6*, and *GA20ox7* were upregulated by both GA_3_ and GA_4_. *GA20ox8* was unique in that it remained completely unaffected. Notably, it was downregulated by decapitation (Figure 4A). Overall, GA feeding showed that the expression of most *GA20ox* genes was under strong homeostatic control. Contrary to the downregulating effect of GA_3_ and GA_4_ at the 3 d time point, GR24 feeding tended to upregulate the expression of several *GA20ox* genes (Figure 6A) whereas at the 5 d time point *GA20ox6*, and *GA20ox7* were significantly upregulated, similarly to GA_3_ and GA_4_.

Of the *GA3ox* family genes, *GA3ox1* was significantly downregulated in AXBs by both GA_3_ and GA_4_ (Figure 6B). That *GA3ox1* expression remained low during the entire period was expected, as it was downregulated by decapitation and did not play a role in AXB activation (Figures 4B, 5B). In contrast, *GA3ox2*, which is characteristically expressed in proliferating apices and upregulated in activated AXBs (Figures 2C, 4B), was very strongly downregulated at day 3, although it recovered at day 5 (Figure 6B). Overall, GA feeding showed that *GA3ox* genes, especially *GA3ox2*, were homeostatically controlled. GR24 did not initially affect the expression of *GA3ox* genes, but at day 5 it significantly increased the expression of the maturation-related *GA3ox1* as well as the proliferation-related *GA3ox2*.

The GA-deactivating *GA2ox* genes were strongly upregulated by both GAs. The GA_4_-induced upregulation of the major *GA2ox1* gene was almost 25-fold at day 3, while GA_3_ was less effective (Figure 6C). In most cases, the expression levels decreased somewhat at day 5. However, *GA2ox5* expression continued to rise during GA_4_ feeding, while this gene was unresponsive to GA_3_. In contrast, the minor genes *GA2ox6* and *GA2ox7*, were more responsive to GA_3_ than to GA_4_ at day 5. The significant upregulation of *GA2ox* genes indicates that both GAs were effective, whereas GR24 had no effect, suggesting that GR24 does not promote GA deactivation in activated AXBs. *GID1* genes were significantly downregulated by GA_3_ and GA_4_ (Figure 6D). Interestingly, GR24 also reduced *GID1* expression almost to the same degree as the GAs, probably because it upregulated many GA biosynthesis genes (Figures 6A,B).

**FIGURE 7:**
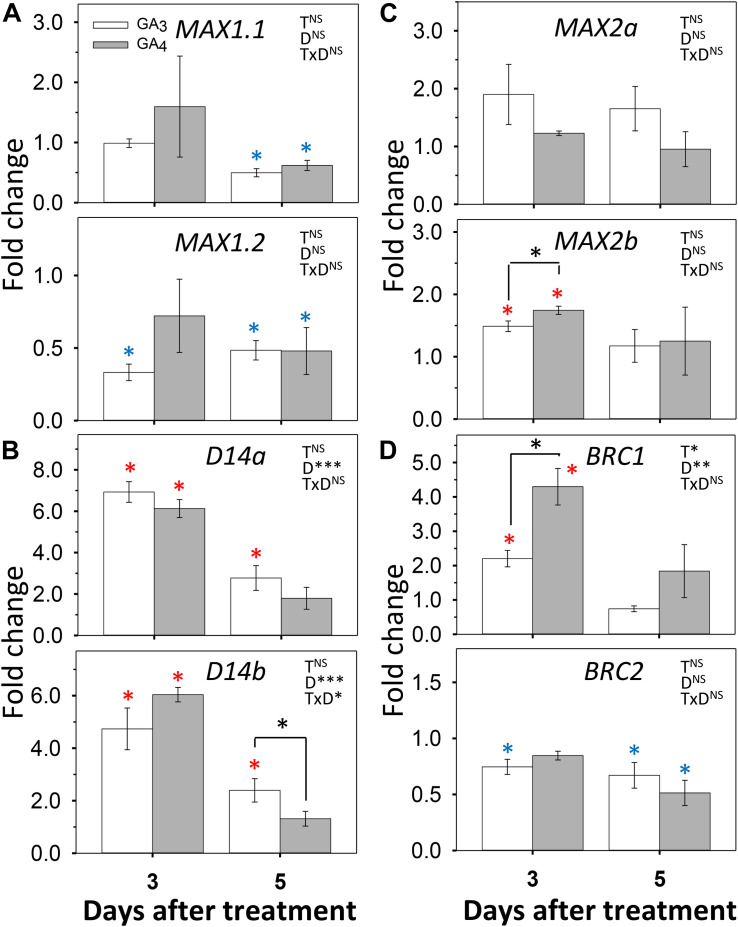
Effect of GA_3_ and GA_4_ on selected SL-pathway genes in AXBs. AXBs on single-node cuttings were fed with or without GA_3_ and GA_4_ at concentration of 10 μM. Gene expression was analyzed after 3 and 5 days of treatment. **(A)** *MAX1.1* and *MAX1.2.* **(B)** *D14a* and *D14b.* **(C)** *MAX2a* and *MAX2b.* **(D)** *BRC1* and *BRC2*. Values are calculated relative to control and represent the means of three biological replicates ± S.E. (*n* = six plants). The significance of factors in two-way ANOVA (T, treatments; D, duration in days; TxD, interaction) are indicated by asterisks (*P < 0.05; **P < 0.01; and ***P < 0.001). Asterisks above the bar indicate decrease (blue) or increase (red), relative to control, and above the hook differences between GA_3_- and GA_4_-treatments (Fischer’s LSD *post hoc* analysis; *P*-value at least < 0.05).

To assess if the reverse could also be the case, we tested how GA_3_ and GA_4_ affected expression of SL pathway genes (Figure 7). In the SL pathway, the gene *MAX1* encodes an enzyme that converts plastid-produced carlactone to the SL precursor carlactonoic acid (Abe et al., 2014). Of the two hybrid aspen paralogs *MAX1.1* and *MAX1.2*, the gene *MAX1.2* was downregulated by GA_3_ and GA_4_, especially by GA_3_, both at day 3 and 5, whereas *MAX1.1* was downregulated only by day 5 (Figure 7A). The genes that encode the SL receptor, *D14a* and *D14b*, were strongly upregulated by GA_3_ and GA_4_, while *MAX2a* and *MAX2b* were moderately upregulated at day 3 (Figures 7B,C). Together this indicates that the GA-induced reduction of SL levels caused upregulation of *D14* and *MAX2* signaling genes through SL homeostasis. This might have transiently increased expression of *BRC1*, a downstream target of SL. In contrast, *BRC2* was slightly downregulated by both GAs (Figure 7D).

### AXB Activation Increases the Ratio of GA_4_/GA_1_ to GA_3_/GA_6_

**FIGURE 8:**
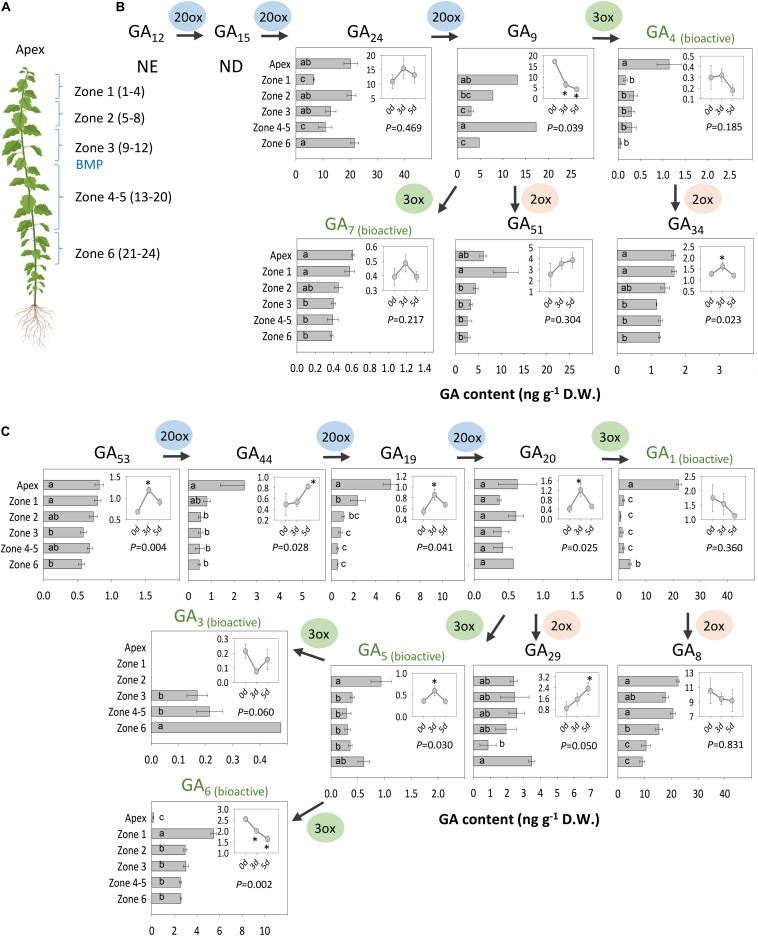
Analysis of GAs. **(A)** Analyzed materials are indicated. The numbers in parenthesis of each zone refer to the position and the number of AXBs in each sample. GAs of the **(B)** non-13-hydroxylation and **(C)** the 13-hydroxylation pathway. Insets: Changes in GA levels 0, 3, and 5 days after decapitation in AXBs proximal to the decapitation point (*P*-value shown). Asterisks in insets indicate statistically significant change in GA levels. Values represent the means of three biological replicates ± S.E. (*n* = six plants). Different letters in bars indicate statistical differences in GA levels between the samples. NE, not estimated; ND, not detected. One-way ANOVA and pairwise *post hoc* analysis by Fischer’s LSD test (*P*-value at least < 0.05).

Gibberellin metabolites, precursors and bioactive molecules in intact and decapitated plants were analyzed using an establish method (Urbanová et al., 2013). This revealed the presence of spatio-temporal patterns in apices and AXBs of distinct zones along the stem (Figure 8). A notable finding was that apices contained bioactive GA of both branches of the GA pathway, although GA_1_ was the dominant bioactive GA in apices, and levels of GA_4_, GA_5_, and GA_7_ were significantly lower, at least by a factor 20. GA_6_ was hardly detectable in apices, whereas GA_3_ was below the detection limit of the LC-MS/MS method used. Although GA_1_ and GA_4_ levels were higher in apices than AXBs, these differences were not reflected at the level of precursors. In the case of GA_4_, its immediate precursor, GA_9_ was under the detection limit in apices, in contrast to GA_24_, which was present at high levels. This could indicate that the pool of GA_9_ is very small due to its rapid conversion to GA_4_, GA_7_ and the deactivation product GA_51_. The GA20ox that produces GA_9_ from GA_24_ could therefore be a rate-limiting enzyme in apices, but not in AXBs where these genes were well expressed. GA_1_ levels in apices were about 40 times higher than the levels of its precursor GA_20_, even though GA_1_ was strongly deactivated to GA_8_. This suggests that the GA_20_ pool is in a state of rapid flux in apices.

In AXBs, GA_1_ levels were ca.10-fold lower than in apices, while the level of the bioactive GA_4_ was about 3- to 4-fold lower (Figures 8B,C). AXBs contained a considerable amount of GA_6_, while GA_3_ was produced at a much lower level, and only in mature AXBs (mature AXBs in zone 4-5) and aging (oldest AXBs in zone 6) (Figures 8A,C). GA-deactivation was especially prominent in the early 13-hydroxylation pathway, resulting in high levels of GA_29_ and, especially, GA_8_. Whereas GA_1_ content was low in AXBs, its deactivation product GA_8_, was almost at the same level as in apices. When the *GA2ox* genes, responsible for this conversion, are abruptly downregulated, as observed after decapitation in AXBs (Figure 4C), GA_1_ availability is expected to rise. In the non-13-hydroxylation pathway, most GA_9_ was de-activated to GA_51_, and comparatively little to the bioactive GA_4_ and GA_7_, both in apices and AXBs. Similarly, to GA_8_, the GA_4_-deactivation product GA_34_ was almost the same in AXBs and apices, suggesting that decapitation-induced downregulation of *GA2ox* expression in AXBs increases GA_4_ availability.

Shoot decapitation only slightly affected GA content during the AXB elongation phase at 3 d and 5 d post-decapitation (Figures 8B,C, insets). The changes in the 13-hydroxylation pathway (Figure 8C) were more often statistically significant than those in the non-13-hydroxylation pathway (Figure 8B). In the latter, only the deactivation product GA_34_ increased significantly. In the 13-hydroxylation pathway, GA_1_ also did not show any increase, even though all its precursors increased at day 3 and 5. The overall increase in precursors (GA_53_ to GA_20_) resulted in a significant increase of the deactivation product GA_29_. GA_3_ and GA_6_ were absent from apices, but were detected in AXBs, whereas decapitation lowered their contents, especially that of GA_6_.

Interestingly, in a separate experiment under suboptimal greenhouse conditions, where plants tended to cease growth, GA_20_ levels and their deactivation products GA_29_ and GA_8_ were higher in apices, while GA_1_ levels were very low (Supplementary Figure S4). In these plants GA_3_ was also detectable in apices, while GA_5_ and GA_6_ were under the detection limit. This highlights that GA_3_ is not unique to AXBs *per se* but can be produced to restrict proliferation.

## Discussion

Shoot branching is regulated by a network of inhibitory and promotive forces. The present results obtained by combining gene expression profiling, metabolite quantitation and hormone treatments show that specific GAs promote branching, while others maintain AXBs in a quiescent state.

### AXB Activation and Outgrowth Require Diminished GA-Deactivation

The differential expression of GA-pathway genes at the whole plant level appears to reflect the proleptic lifestyle of hybrid aspen, in which AXBs become quiescent once they reach maturity (Rinne et al., 2015). AXBs expressed most *GA20ox* genes at significantly higher levels than apices (Figures 2A,B). Nonetheless, the levels of bioactive GA_1/4_ were significantly lower in AXBs than in proliferating apices (Figure 8). The obvious reason for this is that *GA2ox* genes were strongly expressed in AXBs, about 6-fold relative to apices (Figures 2D,E). As the encoded GA2ox enzymes irreversibly deactivate bioactive GAs by 2β-hydroxylation (Thomas et al., 1999; Olszewski et al., 2002; Middleton et al., 2012), the high level of *GA2ox* expression in AXBs can keep them quiescent. This is strongly supported by the fact that during AXB activation several *GA2ox* genes were rapidly and significantly downregulated, and subsequently also the four *GID1* receptor genes (Figures 4C,D). This indicates that GA availability had effectively increased because GID1 levels are known to diminish when GA levels increase due to homeostatic adjustment (Gallego-Giraldo et al., 2008; Hedden and Thomas, 2012; Middleton et al., 2012). Because bioactive GA levels reflect the balance between GA biosynthesis and deactivation (Phillips et al., 1995; Xu et al., 1999; Olszewski et al., 2002; Yamaguchi, 2008), the decapitation-induced reduction of GA deactivation increases its availability for signaling, even in the absence of increased biosynthesis.

**FIGURE 9:**
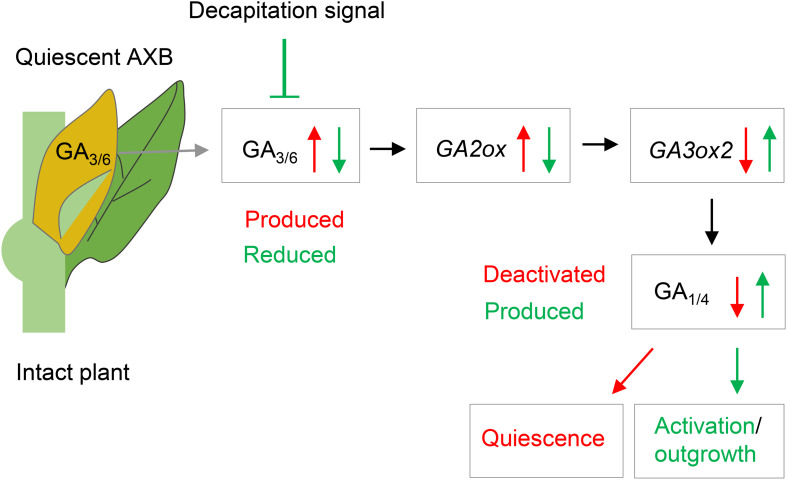
Model of axillary bud (AXB) quiescence and activation. During their formation, AXBs accumulate GA_3/6_, which upregulates *GA2ox* gene expression. GA2ox deactivates GA_1/4_, thereby depriving the AXBs of GA_1/4_-mediated signaling. Decapitation activates quiescent AXBs by rapidly reducing GA_3/6_ levels and downregulating *GA2ox* transcription, thereby elevating GA_1/4_ levels and signaling. Subsequently, the GA_1/4_ biosynthesis gene *GA3ox2* is upregulated dramatically increasing GA levels and promoting AXB outgrowth. Red arrows and text indicate AXB inhibitory effects. Green arrows and text indicate AXB activating and growth promoting effects.

The emerging picture is that quiescent AXBs are sensitized to GA, because relative to apices they have low levels of GA_1_ and GA_4_ despite the ongoing GA biosynthesis, but high levels of *GID1* expression. Thus, regardless of GA biosynthesis, the dwarfed ES of AXBs is GA deficient. The high *GA2ox* expression levels in AXBs appear to be developmentally controlled to keep AXB activation at bay and safeguard the proleptic nature of the shoot system. GA_3/6_ can play a role in maintaining AXB quiescence (Figure 8C) by upregulating *GA2ox* genes, thereby deactivating GA_1/4_, but not of itself (and GA_5/6_) because it is not a substrate (Nakayama et al., 1990; Ito et al., 2017; Li et al., 2017). Thus, the specific presence of GA_3/6_ in quiescent AXBs can effectively maintain them in a GA_4_-deficient state. As GA_4_ is involved in promoting cell division, elongation and energy metabolism (Hedden and Sponsel, 2015; Zhuang et al., 2015) and has the highest binding activity to GID1 (Ueguchi-Tanaka et al., 2005), keeping GA_4_ low is necessary to prevent AXB activation and outgrowth. In addition, other factors may play a role in AXB quiescence, including SL (Katyayini et al., 2019) and BRC1-regulated ABA signaling (González-Grandío et al., 2017; Wang et al., 2019). After AXB activation, subsequent AXB elongation is supported by *de novo* biosynthesis of GA_1_ and GA_4_, initiated between 12 and 24 h through upregulation of *GA3ox2* (Figure 4B). In support of this, a previous study showed that this gene, originally named *GA3ox1*, is characteristically expressed in growing shoot apices (Israelsson et al., 2004). In short, our data support a model in which branching is initiated by a strong reduction of GA deactivation that raises the bioactive GA_1/4_ pool to spearhead AXB activation, while additional GA_1/4_ biosynthesis supports subsequent AXB elongation, as illustrated in Figure 9.

### GA Biosynthesis Differs in Growing and Mature Tissues

The expression patterns of the GA biosynthesis genes were different for actively proliferating tissues (apices and roots), differentiated tissues (mature leaves), and developmentally inactive tissues with high growth potential (AXBs) (Figure 2). For example, apices expressed *GA20ox* genes less than other tissues, but they highly expressed *GA3ox2*, whereas *GA3ox1* was hardly expressed. In contrast, quiescent AXBs expressed both *GA3ox* genes, whereas source leaves exclusively expressed *GA3ox1* genes. Thus, *GA3ox2* supports cell proliferation and growth at apices and root tips, whereas *GA3ox1* reflects tissue maintenance in source nodes and leaves. The fact that quiescent AXBs expressed both *GA3ox2* and *GA3ox1* appears to reflect their opposing developmental tendencies, as AXBs combine developmental stasis with high growth potential. As indicated above, the high levels of GA deactivation, maintained by the GA2ox-insensitive GA_3/6_, are likely to be part of the developmental block that prevents AXB activation.

Although AXBs expressed all GA-pathway genes, their outgrowth is strongly dependent on a functional connection to the stem, especially nodal vascular tissue. The results suggest that nodal bark exported precursors to AXBs, because the *GA20ox* transcript levels in the AXBs were reduced soon after decapitation, whereas in the nodal bark they initially increased without increasing *GA3ox2* expression (Figures 5A,B). Transport of precursors and bioactive GAs (GA_3_, GA_4_, GA_9_, GA_12_ and GA_20_) is known to be crucial in directing development (Proebsting et al., 1992; Eriksson et al., 2006; Yamaguchi, 2008; Ragni et al., 2011; Dayan et al., 2012; Lange and Lange, 2016; Regnault et al., 2016; Binenbaum et al., 2018). The GA quantitation data support the idea that precursors are transported from nodes to the AXBs, as their levels increased in AXBs after decapitation, for example in case of GA_20_, a key precursor of several bioactive forms of GA (Figure 8C). Such node-to-AXB delivery also plays a role in the SL-mediated control of AXB quiescence (Katyayini et al., 2019). Together, the analyses indicate that nodal bark tissue might affect AXBs by delivering SL and GA precursors.

### GA and SL Pathways Are Buffered and Show Interference

During the AXB elongation phase, *GA2ox* genes responded strongly to GA feeding by upregulating their expression up to ≥20-fold at day 3. As the *GID1* expression levels were only reduced by about 2-fold, the upregulated *GA2ox* must have been effective in deactivating part of the supplied GA. Feeding GR24 did not affect the expression of *GA2ox* genes, but it did increase the expression of GA biosynthesis genes at day 5 (Figures 6A,B). A putative increase in GA levels by GR24 could explain why GR24 feeding reduced *GID1* expression levels to a similar degree as GA_3_ and GA_4_ (Figure 6D).

In hybrid aspen, SL pathway and perception genes are highly expressed in quiescent AXBs, but decapitation rapidly downregulated these genes as well as the downstream target gene *BRC1* (Katyayini et al., 2019). While GA_3/6_, GA2ox as well as SL contribute to the quiescent state of AXBs in intact plants, their decrease in activated AXBs leads to elevated GA_1/4_ levels through a reduction of GA2ox activity. Subsequent outgrowth might require CK in addition (Ni et al., 2017; Duan et al., 2019).

As feeding GA_3_ and GA_4_ reduced the expression of both *MAX1* genes (Figure 7A), GA represses SL biosynthesis, which supports earlier observations in other plant species (Ni et al., 2015; Ito et al., 2017; Marzec, 2017). Our data show that during the AXB elongation phase both GA_3_ and GA_4_ increased SL perception by upregulating *D14* genes and *MAX2b* (Figures 7B,C). This increase in SL perception and signaling genes presumably is a homeostatic response to a GA-induced reduction in SL levels in the AXBs. In Arabidopsis, GA and GR24 converge on a large number of shared transcription targets (Lantzouni et al., 2017). However, in pea, SL can also independently of GA promote cell division in the stem (de Saint Germain et al., 2013). Here we found that GR24 increased the biosynthesis of GA during the AXB elongation phase. It is noteworthy that GR24 feeding can promote the elongation of the enclosed ES five to seven days post-decapitation (Katyayini et al., 2019), and the present data suggest this might involve GA. Whether these interferences between SL and GA pathways are direct or indirect remains to be established.

### GA_3_ and GA_6_ Are Involved in AXB Development but Not in AXBs Outgrowth

In AXBs of intact plants, the gene *GA3ox1* could be linked to presence of GA_3_ and GA_6_. After decapitation, *GA3ox1* expression and GA_3_ and GA_6_ content decreased in AXBs (Figures 4B, 8C) and were absent from apices (Figure 2C). This indicates that *GA3ox1* functions in the side branch of the 13-hydroxylation pathway that produces the deactivation-protected GA_3_, GA_5_ and GA_6_. In contrast, *GA3ox2* converts precursors GA_9_ and GA_20_ to GA_7_, GA_4_ and GA_1_, in support of a previous study (Israelsson et al., 2004).

In apices GA_1_ was more abundant than GA_4_ (Figure 8), although GA_4_ more efficiently promotes shoot elongation (Israelsson et al., 2004). However, plants can switch between pathways, depending on developmental phase or environmental conditions (Rieu et al., 2008b). For example, in a grass species GA_4_ is produced during vegetative growth, while upon flowering it switched to GA_5_ and GA_6_ (King et al., 2001, 2003). That *GA2oxs* play a role in this, is supported by studies in *Jatropha*, where overexpression of *GA2ox6* induced a switch from the non-13-hydroxylation pathway (GA_4_) to the 13-hydroxylation pathway (GA_3_), and led to dwarfing (Hu et al., 2017). Our data suggest that the GA precursor GA_20_ can be converted to the growth-promoting GA_1_ or the quiescence-related GA_3/6_ (Figure 8C) dependent on developmental cues as well as environmental conditions. GA_3_ accumulates in developing AXBs as well as in apices of stressed plants, while GA_1_ levels remain low (Supplementary Figure S4). The effect of these cues on GA metabolism, and the distinct responses of plants to different bioactive GAs (Elfving et al., 2011; Rinne et al., 2011; Ni et al., 2015) warrant further investigation.

Although GA_3_ is often used as a generic GA, it is different from GA_4_ in important respects. The results show that in hybrid aspen GA_3_ and GA_4_ not only operate at distinct locations, their functions are also partly distinct. GA_4_ feeding promotes AXB outgrowth, whereas GA_3_ induces abscission in the non-dormant quiescent AXBs that form under long days (Supplementary Figure S3) as well as the AXBs that establish dormancy under short days (Rinne et al., 2011). GA_3_ and GA_4_ also induce different classes of 1,3-β-glucanases, destined for different subcellular locations (Rinne et al., 2011). Both GA_3_ and GA_4_ promote cell division, but GA_4_ function requires histone deacetylases to transcriptionally block *GA2ox* (Li et al., 2017). Although required for apical growth, in the vegetative in the meristem dome itself GA_4_ is absent, because its production is blocked, and a band of *GA2ox* expression below the meristem protects it from a damaging influx of GA_4_ (Sakamoto et al., 2001; Jasinski et al., 2005; King et al., 2008; Bolduc and Hake, 2009). As GA_3_ cannot be deactivated by GA2ox, GA_3_ (as well as GA_5_ and GA_6_) can enter the meristem and induce floral transition in grasses, whereas GA_4_ can only enter later, when the band of *GA2ox* expression is gone (King et al., 2003).

Because GA_3_ can significantly upregulate *GA2ox* genes (Figure 6C), its accumulation in quiescent AXBs results in low levels of GA_1/4_ due to deactivation, as both are substrates of GA2ox (Nakayama et al., 1990), thereby inhibiting GA_4_-mediated AXB activation and elongation. Our finding that GA_3/6_ were detected in quiescent AXBs and reduced by decapitation, matches our earlier finding that GA_3_, unlike GA_4_, cannot upregulate the growth-related α-clade 1,3-β-glucanases that optimize symplasmic conduits for transport to growing areas (Rinne et al., 2011).

## Conclusion

A major finding was that hybrid aspen invests energy into producing and simultaneously deactivating GA_1/4_ in quiescent AXBs, although they remain developmentally inactive until the next year. This seemingly wasteful strategy is an effective way to keep AXBs ready for rapid outgrowth in case the shoot apex is damaged or lost, allowing a new shoot to form before winter arrives. The results support a model in which SL and *GA3ox1-*mediated accumulation of GA_3/6_ maintain AXBs in a quiescent state, with GA_3/6_ upregulating *GA2ox* genes that deactivate GA_1/4_. In turn, decapitation-induced AXB activation is triggered by a rapid downregulation of *GA2ox* genes, which shifts the balance between GA_1/4_ biosynthesis and deactivation, increasing the GA_1/4_ pool available for GA signaling. The initial GA_1/4_ pulse is followed by increased *GA3ox2*-mediated *de novo* GA biosynthesis, and subsequent elongation of the AXB. The dual, opposing roles of GA_3/6_ and GA_1/4_ can explain why the role of GA in branching has been ambiguous.

## Materials and Methods

### Plant Material and Sample Preparation

Hybrid aspen (*Populus tremula* × *Populus tremuloides*) clone T89 was micro-propagated *in vitro* and grown in a greenhouse under long days as previously described (Katyayini et al., 2019). When the plants were 80–100 cm tall, with stable leaf production and elongation rates, they were subdivided into three groups: (a) Intact plants for collection of tissues and organs for transcript analyses; (b) Decapitated plants (decapitated at the bud maturation point, ca. 40 cm below the apex), for transcript and GA analysis in AXBs, and transcript analysis of nodal bark; (c) Plants for xylem feeding of hormones into single-node cuttings. Samples for transcript and hormone analyzes were collected from six plants, with two plants pooled in three replicate samples. Position of sampled buds and tissues is indicated in Supplementary Figure S5.

### Quantification of GAs With Liquid Chromatography-Mass Spectrometry (LC-MS/MS)

The samples (apices and AXBs) were harvested from different zones along the stem, as indicated in Figure 3A. For analysis, samples were immediately frozen in liquid nitrogen, and subsequently freeze dried. Sample preparation and quantitative analysis of GAs were performed by LC-MS/MS using ^2^H_2_-labeled GA internal standards as described (Urbanová et al., 2013).

### AXB Burst Tests and Feeding of GA_3_, GA_4_, and GR24

To investigate the effects of GA_3_, GA_4_ and the synthetic strigolactone GR24 on AXB outgrowth and gene expression, we performed xylem-feeding experiments under forcing conditions in growth chambers (18 h of light with a PPFD of 160−200 μmol m^–2^ s^–1^, 20°C, and 60% relative humidity). Single-node cuttings were isolated from 6-week old plants. The internode base was punched through pores in a Styrofoam sheet, floated on water (control) or water supplemented with GA_3_, GA_4_ (Sigma-Aldrich) or racemic synthetic SL GR24 (Chiralix BV, Netherlands) at the effective 10 μM concentration (Katyayini et al., 2019). AXB burst was followed for 14 days and scored as Σ_14_-values, as explained in Supplementary Figure S3.

### Experiment Design and Gene Selection

For analysis of GA-pathway, total RNA was extracted from different plant parts as indicated (Figure 2). Gene expression analysis included hybrid aspen homologs of *P. trichocarpa* GA-biosynthesis genes *GA20ox2-1*, *GA20ox3*, *GA20ox4, GA20ox5, GA20ox6, GA20ox7*, *GA20ox8, GA3ox1*, and *GA3ox2*; GA-catabolism genes *GA2ox1*, *GA2ox3*, *GA2ox4*, *GA2ox5*, *GA2ox6*, and *GA2ox7*; GA-signaling genes *GID1A-1*, *GID1A-2*, *GID1B-1*, and *GID1B-2*. For phylogenetic analysis, see Supplementary Figures S1, S2.

To assess decapitation-induced expression changes, AXBs proximal to the decapitation point of the BMP were collected 0, 2, 6, 12, 24, and 48 h post-decapitation. Sampling after day 1 and day 2 was carried out at the same time of day to avoid potential diurnal effects on gene expression. Nodal bark tissues were collected 0, 2, 6, and 12 h after decapitation.

The effects of 10 μM GA_3_, GA_4_ and GR24 on gene expression in AXBs were investigated after xylem feeding of the hormones into AXBs of single-node cuttings. Samples were collected after 0, 3, and 5 days. Gene expression analysis included GA-biosynthesis *GA20ox2-1, GA20ox3*, *GA20ox4*, *GA20ox6, GA20ox7*, *GA20ox8, GA3ox1*, and *GA3ox2*; GA-catabolism genes *GA2ox1*, *GA2ox3*, *GA2ox4*, *GA2ox5*, *GA2ox6*, and *GA2ox7*; GA-signaling genes *GID1A-1*, *GID1A-2*, *GID1B-1* and *GID1B-2*. In addition, previously identified SL-biosynthesis and signaling genes *MAX1.1*, *MAX1.2*, *D14a*, *D14b*, *MAX2a*, and *MAX2b*, and the downstream target genes *BRC1* and *BRC2* (Katyayini et al., 2019) were analyzed after GA_3_ and GA_4_ feeding.

### RNA Extraction and cDNA Preparation

Total RNA was extracted from 0.2 to 0.3 g of frozen tissue and grinded in a mortar with 500 μL extraction buffer (Qiagen RLT buffer containing 1% PVP-40), and further processed as described (Katyayini et al., 2019). The samples were transferred to RNeasy spin columns and further processed in accordance with instructions of the Qiagen Plant RNA isolation kit. Genomic DNA was eliminated using TURBO^TM^ DNase kit (Invitrogen) treatment according to manufacturer’s instructions and cleaned using the total RNA purification system “Purelink RNA mini kit” (Invitrogen). RNA was quantified with NanoDrop 1000, and the RNA quality was assessed with the Agilent 2100 Bioanalyzer system. 1 μg of total RNA was reversely transcribed to cDNA with SuperScript^®^ VILO^TM^ reverse transcriptase (Invitrogen).

### Quantitative RT-PCR (qRT) Analysis

The reaction setup (20 μl total volume) for qRT was prepared using SYBR^®^ select PCR master mix (Applied Biosystems). As a template, 2 μl of the cDNA (200 ng) were added. Real-time qRT-PCR analyses were performed with the Applied Biosystems 7500 Fast Real-Time PCR system according to the manufacturer’s instruction. Thermocycling conditions were set to 50°C for 2 min, 95°C for 2 min, 45 cycles of 15 s at 95°C and 60 s at 60°C. Each PCR reaction included a negative control to check for potential genomic DNA contamination. For a complete list of primers and genes used for quantitative real time PCR (qRT-PCR) see Supplementary Table S1.

### Statistical Analysis and Bioinformatics

Statistical analyses were carried out using analysis of variance (one- or two-way ANOVA) in combination with Fisher LSD *post hoc* test to determine significant differences between the subgroups. Computation was performed using Microsoft Excel data analysis^1^ and Minitab Statistical Software version 18.1.^2^

BLAST searches in GenBank, *Populus trichocarpa* genome v3.0 and *Populus tremula* × *Populus tremuloides* (T89) v3.0 databases^3, 4, 5^ were used to identify GA-biosynthesis, -catabolism and -signaling genes. Gene specific primer sequences for qPCR analysis were designed using Primer3.^6^

## Data Availability Statement

All datasets generated for this study are included in the article/Supplementary Material.

## Author Contributions

NK, PR, and CS designed the research. NK, PR, and DT performed the experiments. NK, PR, and CS analyzed and interpreted the data. NK designed the illustration. All authors participated in writing and revising the manuscript.

## Conflict of Interest

The authors declare that the research was conducted in the absence of any commercial or financial relationships that could be construed as a potential conflict of interest.

## Footnotes

1 www.microsoft.com

2 www.minitab.com

3 http://www.ncbi.nlm.nih.gov

4 http://www.phytozome.net

5 http://popgenie.org

6 http://bioinfo.ut.ee/primer3-0.4.0
