# Supplementary material for: Dual Role of Gibberellin in Perennial Shoot Branching: Inhibition and Activation
Source: Front Plant Sci. 2020 Jun 5;11:736. doi: 10.3389/fpls.2020.00736 (PMC7289990; doi:10.3389/fpls.2020.00736)
Supplement: Supplementary file 1 [file Data_Sheet_1.PDF]

frontiers

Supplementary Material

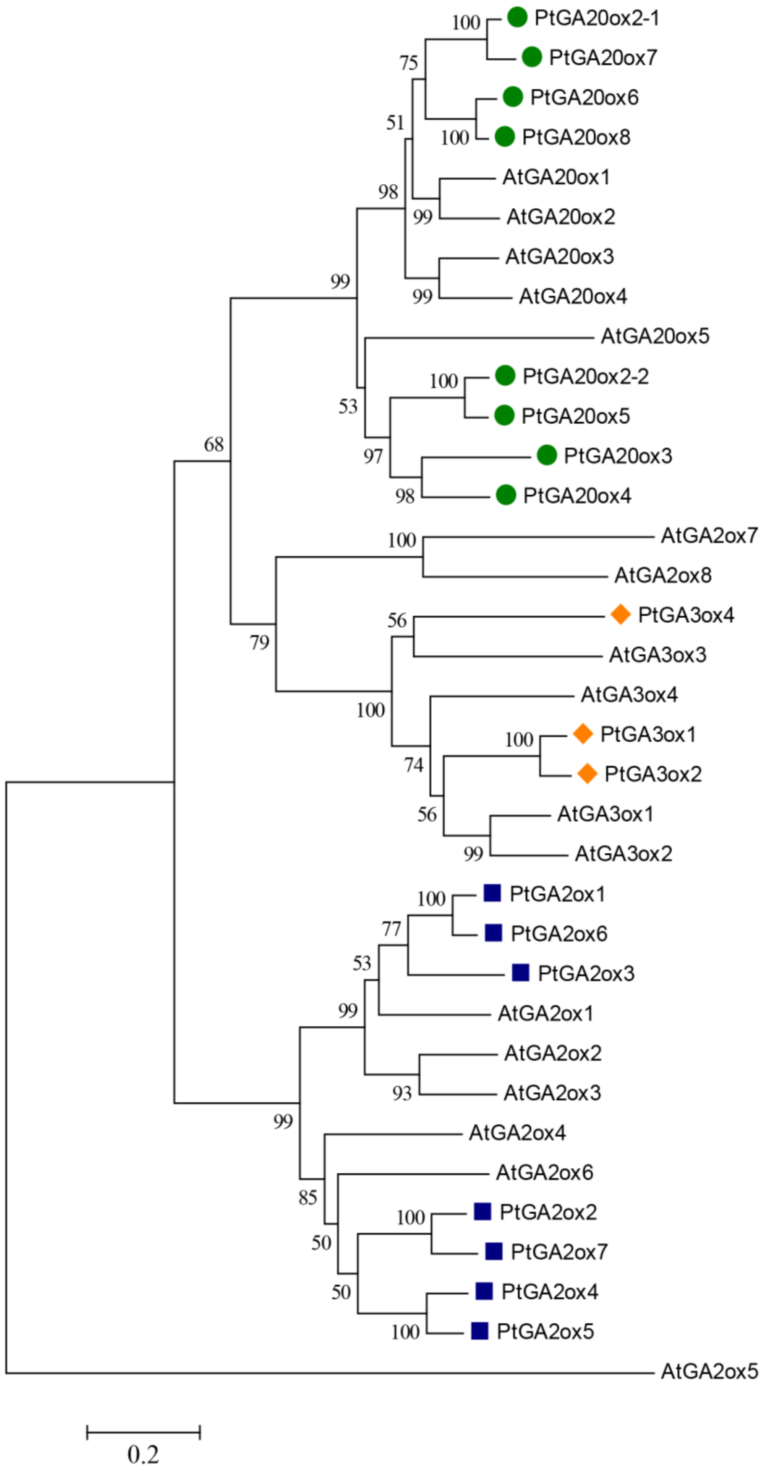

Supplementary Figure 1. Phylogenetic analysis of GA20-oxidases, GA3-oxidases and GA2-oxidases. The sequence homologues were identified by Protein BLAST search in NCBI

(http://www.ncbi.nlm.nih.gov/BLAST) and the sequences were retrieved from *Populus trichocarpa* genome (Tuskan *et al.*, 2006; http://www.phytozome.net/) databases. The amino acid sequence alignment were performed using ClustalW (http://www.ebi.ac.uk/Tools/msa/clustalw2). A phylogenetic tree was created using the MEGA6 program (www.megasoftware.net) with the Neighbor–Joining method. Bootstrap support values are based on 1000 replicates. The proteins used in the phylogenetic analysis were: *Arabidopsis thaliana* AtGA2ox1 (At1g78440), AtGA2ox2 (At1g30040), AtGA2ox3 (At2g34555), AtGA2ox4 (At1g47990), AtGA2ox5 (At3g17203), AtGA2ox6 (At1g02400), AtGA2ox7 (At1g50960), AtGA2ox8 (At4g21200), AtGA20ox1 (At4g25420), AtGA20ox2 (At5g51810), AtGA20ox3 (At5g07200), AtGA20ox4 (At1g60980), AtGA20ox5 (At1g44090), AtGA3ox1 (At1g15550), AtGA3ox2 (At1g80340), AtGA3ox3 (At4g21690), AtGA3ox4 (At1g80330), AtGID1a (At3g05120), AtGID1b (At3g63010), AtGID1c (At5g27320); *Solanum lycopersicum* SlGID1a (Solyc01g098390), SlGID1b1 (Solyc09g074270), SlGID1b2 (Solyc06g008870); *Oryza sativa* OsGID1 (LOC_Os05g33730); *Hordeum vulgare* HvGID1 (A7MAQ4); *Populus trichocarpa* PtGA2ox1 (Potri.001G378400), PtGA2ox2 (Potri.002G191900), PtGA2ox3 (Potri.004G065000), PtGA2ox4 (Potri.008G101600), PtGA2ox5 (Potri.010G149700), PtGA2ox6 (Potri.011G095600), PtGA2ox7 (Potri.014G117300), PtGA20ox2-1 (Potri.002G151300), PtGA20ox2-2 (Potri.005G065400), PtGA20ox3 (Potri.005G184400), PtGA20ox4 (Potri.005G184200), PtGA20ox5 (Potri.007G103800), PtGA20ox6 (Potri.012G132400), PtGA20ox7 (Potri.014G073700), PtGA20ox8 (Potri.015G134600), PtGA3ox1 (Potri.001g176600), PtGA3ox2 (Potri.003g057400), PtGA3ox4 (Potri.018G033600). PtGA20oxs (●); PtGA2oxs (◆); PtGA3oxs (■).

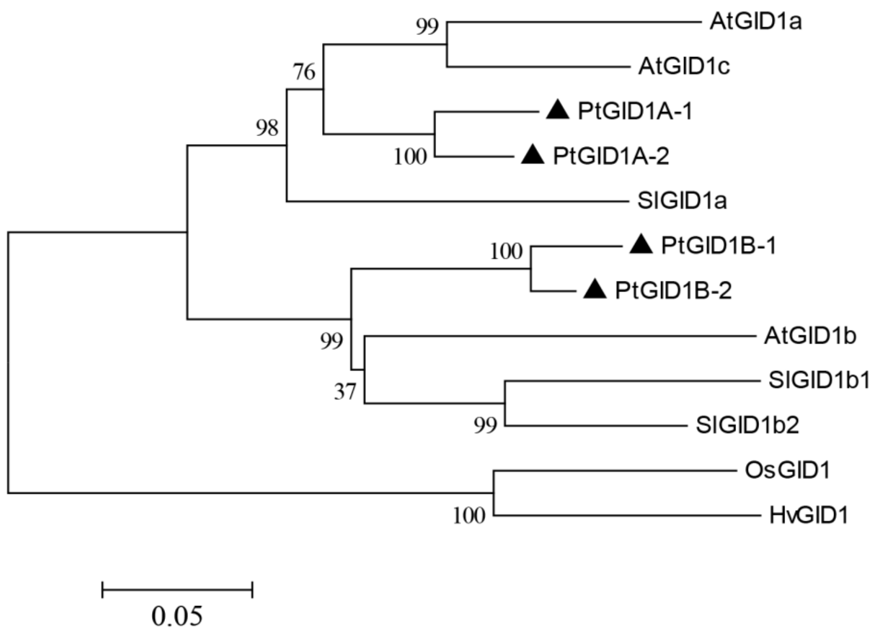

Supplementary Figure 2. Phylogenetic analysis of GID1, gibberellin receptor proteins. The sequence homologues were identified by Protein BLAST search in NCBI (http://www.ncbi.nlm.nih.gov/BLAST) and the sequences were retrieved from *Populus trichocarpa* genome (Tuskan *et al.*, 2006; http://www.phytozome.net/) databases. The amino acid sequence alignment were performed using ClustalW (http://www.ebi.ac.uk/Tools/msa/clustalw2). A phylogenetic tree was created using the MEGA6 program (www.megasoftware.net) with the Neighbor–Joining method. Bootstrap support values are based on 1000 replicates. The proteins used

in the phylogenetic analysis were: *Arabidopsis thaliana* AtGID1a (At3g05120), AtGID1b (At3g63010), AtGID1c (At5g27320); *Solanum lycopersicum* SlGID1a (Solyc01g098390), SlGID1b1 (Solyc09g074270), SlGID1b2 (Solyc06g008870); *Oryza sativa* OsGID1 (LOC_Os05g33730); *Hordeum vulgare* HvGID1 (A7MAQ4); *Populus trichocarpa* PtGID1A-1 (Potri.005G040600), PtGID1B-1 (Potri.014G135900), PtGID1A-2 (Potri.013G028700), PtGID1B-2 (Potri.002G213100). PtGID1s (▲).

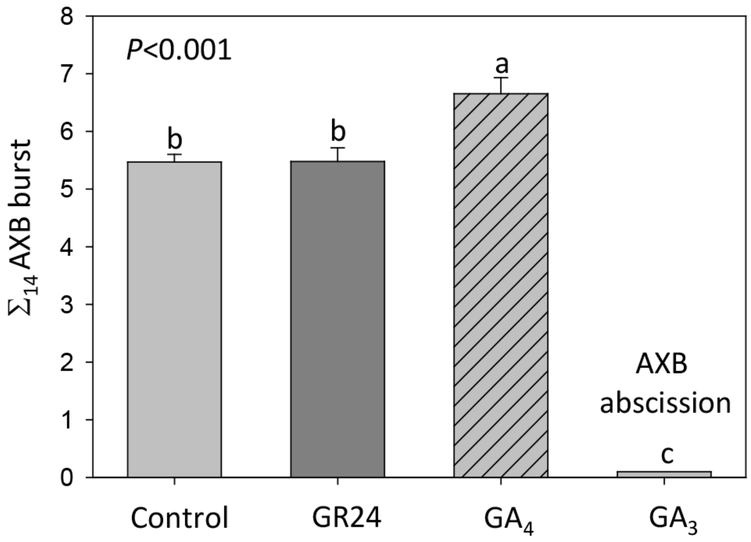

Supplementary Figure 3. Effect of hormones on AXB outgrowth. AXB outgrowth was studied in single node systems xylem-fed with or without 10µM GR24, GA_3 or GA_4. Σ_{14} values refer to timing of AXB burst. If AXB burst early, for example on day 1, AXB scores 14, if on day 14, score is 1. The values are means of two AXBs of 12-16 plants per treatment. One-way ANOVA (*P*-value). Different letters indicate statistical significance between the treatments (Fischer's LSD *post hoc* analysis; *P*-value at least <0.05).

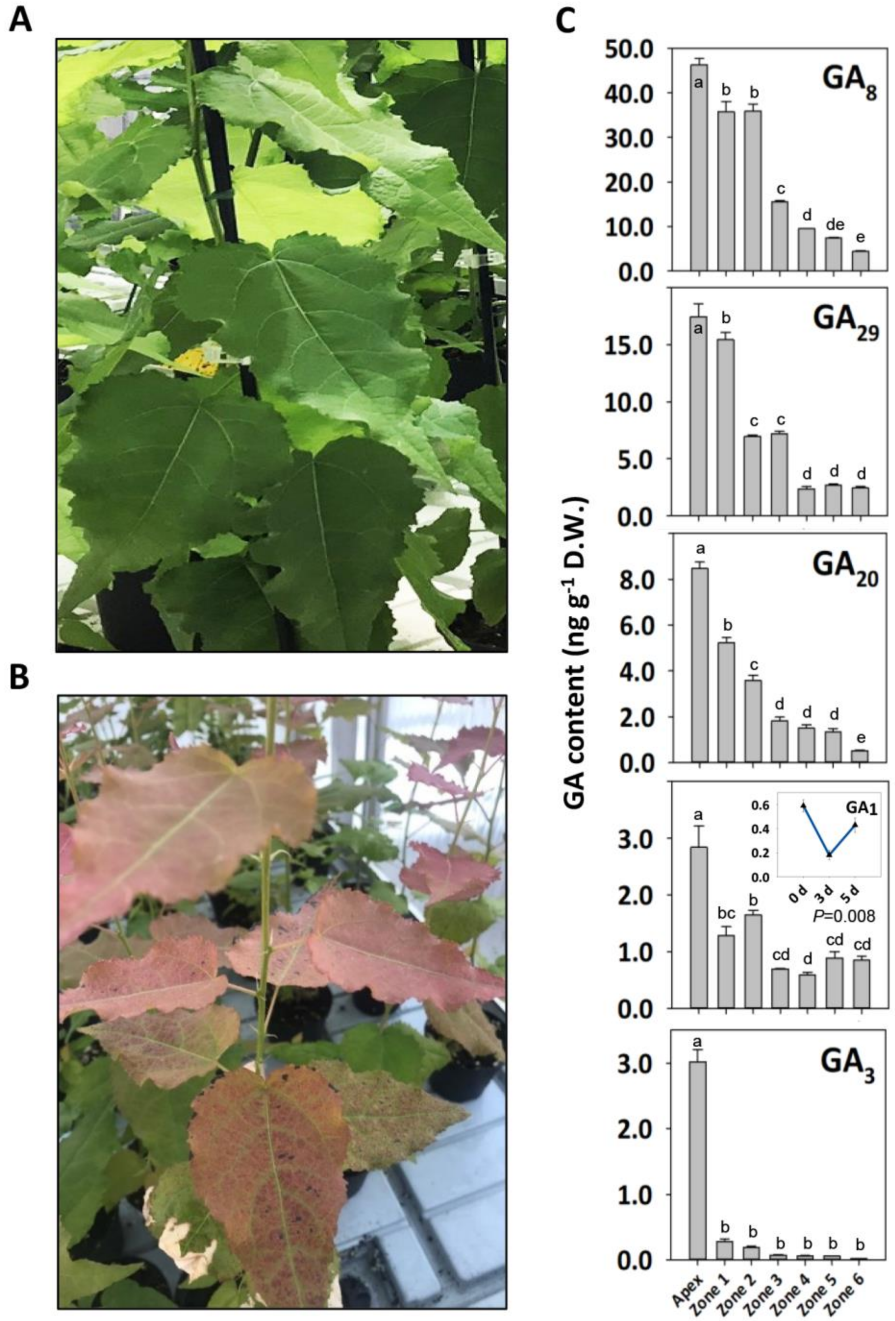

Supplementary Figure 4. GA pathway switches to 13-hydroxylation under suboptimal environmental conditions. (A) Plants grown in optimal conditions (see analysis in Figure 8). (B) Red color as indicator of light stress. (C) GA analysis of plants shown in (b). Inset in GA_1: Changes in GA levels 0, 3 and 5 days after decapitation in AXBs proximal to the decapitation point (*P*-value shown). Asterisks in insets indicate significance change in GA levels. Different letters in bars indicate statistical differences in GA level between the samples. One-way ANOVA and pairwise *post hoc* analysis by Fischer's LSD test (*P*-value at least <0.05).

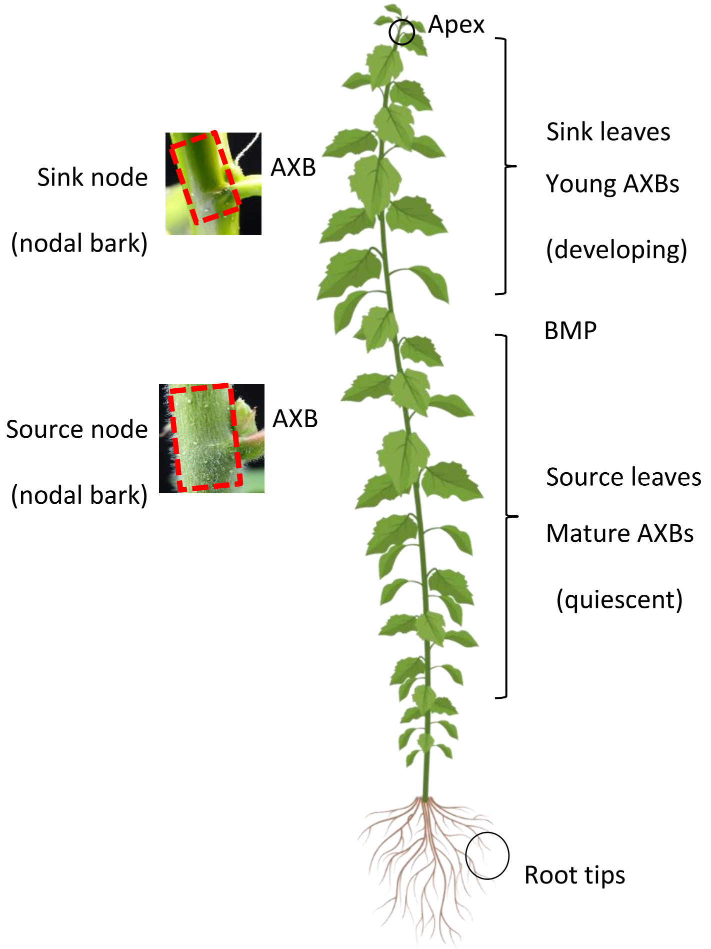

Supplementary Figure 5. Sampled materials. Young axillary buds in axils of sink leaves are associated with sink nodes, whereas mature buds in the axils of source leaves are associated with source nodes. Nodal bark of sink and source nodes was peeled off (stippled red box) under a dissection microscope, followed by analysis. BMP, bud maturation point; AXB, axillary bud.

Supplementary Table 1. ***P. trichocarpa*** **genes, identifiers and primer pairs used for qPCR analysis.**

Populus trichocarpa					
**Protein**	**Gene abb.**	**Locus name v2.0**	**Locus name v3.0**	**Forward 5′-3′**	**Reverse 5′-3′**
**Gibberellin Biosynthesis genes**					
GA3 oxidase1	*GA3ox1*	POPTR_0001s17680	Potri.001G176600	TGGCTCTCCTCTTGAGCATT	AACCATGTCAACCTCCTTG
GA3 oxidase2	*GA3ox2*	POPTR_0003s05610	Potri.003G057400	CCCTATCTCGCTCAATCTTTCC	AGTCAAGGTGCTTTTGGTGTAG
GA20 oxidase2-1	*GA20ox2-1*	POPTR_0002s15260	Potri. 002G151300	CGAAAAACCATGCCTTGAAT	GCCAAAGGATCTCCAGTGAG
GA20 oxidase3	*GA20ox3*	POPTR_0005s20660	Potri.005G184400	TCGGATCTCGTTGTGCTAGA	AGTTCCAATATGGCGAAGGA
GA20 oxidase4	*GA20ox4*	POPTR_0005s20650	Potri. 005G184200	GGCAATAAAGCAGGCTTCTG	TGTGATCATGGGCGAGACTA
GA20 oxidase5	*GA20ox5*	POPTR_0007s04360	Potri. 007G103800	AGCTTGCCCACAGAGTTCAT	GAGCAGTTGCAACCTCATCA
GA20 oxidase6	*GA20ox6*	POPTR_0012s14040	Potri.012G132400	ATTTCGACGCTTTTGTCGTT	GAGATTTTCTTGGCGTTTGG
GA20 oxidase7	*GA20ox7*	POPTR_0014s06960	Potri.014G073700	ATGGCACTCCGTTACTCCTG	CCACTGCTCTATGCAAGCAA
GA20 oxidase8	*GA20ox8*	POPTR_0015s14030	Potri.015G134600	ATCAAAACCATGCCATCCA	TGGTGTCGAAGAACTTGTGC
**Gibberellin deactivation genes**					
GA2 oxidase1	*GA2ox1*	POPTR_0001s38760	Potri.001G378400	TTCTTCTCATTACCGCTCTCTG	TCTACCCAGCCCACATCAC
GA2 oxidase2	*GA2ox2*	POPTR_0002s19260	Potri.002G191900	TGCCTTCCAGGTTTTAACGA	GGCAAGACCAGCTGTGGAG
GA2 oxidase3	*GA2ox3*	POPTR_0004s06380	Potri.004G065000	GGACCTCCTAACCCTTTTGG	TGGGTTTTCCTGAAAAATGG
GA2 oxidase4	*GA2ox4*	POPTR_0008s10100	Potri.008G101600	AGGTAGGGTTCGGAGAGCAT	GGTAGCGGGATCAGGTGTTA
GA2 oxidase5	*GA2ox5*	POPTR_0010s15950	Potri.010G149700	AATGGCCTATTTTGCTGCAC	TATCTCCAAGTCGCAGAGCA
GA2 oxidase6	*GA2ox6*	POPTR_0011s09770	Potri.011G095600	CAAGCCAGCACTTCAACAGT	ATTCCTCACATGCCTTGACC
GA2 oxidase7	*GA2ox7*	POPTR_0007s04360	Potri.014G117300	TTGCTTGCATGATGGTTTGT	GCCTCACGCTTTCAAATCTC
**Gibberellin signaling genes**					
GIBBERELLIN INSENSITIVE DWARF1	*GID1A-1*	POPTR_0005s04240	Potri.005G040600	ACCGTGGGACTAGCCTTCTT	ACAACCTCCGAGTTGACAGG
GIBBERELLIN INSENSITIVE DWARF1	*GID1B-1*	POPTR_0014s13170	Potri.014G135900	GATCATGTTGATCGCACCAC	GTGCTCAAGGGCTTTTCAAG
GIBBERELLIN INSENSITIVE DWARF1	*GID1A-2*	POPTR_0013s02980	Potri.013G028700	GGACCGAGATTGGTACTGGA	TAAACCAGCCACCACAACAA
GIBBERELLIN INSENSITIVE DWARF1	*GID1B-2*	POPTR_0002s22840	Potri.002G213100	GGGGAAAAAGCTTGAAGGAC	CAATTGCCAGTCTTGAACGA
**Housekeeping gene**					
ACTIN	*ACT*	POPTR_0001s31700	Potri.001G309500	CGATGCCGAGGATATTCAAC	ACCAGTGTGTCTTGGTCTACCC
